# Overcoming the laws of the jungle: a multicomponent arts therapy approach for traumatic grief treatment

**DOI:** 10.1192/j.eurpsy.2026.11740

**Published:** 2026-07-31

**Authors:** S. M. Andrei, M. Cassen, B. Raz

**Affiliations:** 1 IFATC, Lyon; 2 Institut Michel Montaigne, Cenon; 3 Family Therapy, Centre Hospitalier de Roanne, Roanne, France

## Abstract

**Introduction:**

Benefits of the use of musical and cinematic arts in psychotherapy have been documented in some settings ever since the seventies, as reviewed in a large meta-analysis by E. Sacilotto et.al (2022, Front. Psychol. 12:732246). Lately it was described as a mental health tool (cinematherapy) for depression (A.Pannu et. al, J.Psychol. 2025;159:329-357), but its potential for treating various psychiatric difficulties is still unfolding. From a scientific point of view, the use of image and sounds brings multidimensional access to both cortical and limbic brain structures, creating a powerful setting for therapeutic change. The additional usage in our model of psychodrama techniques facilitates emotional processing, engaging resources towards growth.

**Objectives:**

This model addresses traumatic grief both in a family therapy setting, and in psychotherapy training.

**Methods:**

Artistic, systemic and trauma-informed approaches were used in the process. For adressing the subject of grief, the choice was the “Lion King” animation movie (1994). The sessions started with musical elements created by Hans Zimmer, as the exposure to prosocial songs is thought to increase accessibility to helping behavior, mediated by empathy. Then specific elements from the movie were presented to introduce safely, piece by piece, the difficult themes of prolonged traumatic grief. Further cinematic elements were carefully chosen to shed light to outcome versions of growth according to characters’ styles and choices.

In a second phase, through psychodrama techniques (enactment of Simba and his family in the therapy room), the therapist accompanies the family through the process of witnessed grief, re-remembering and restructuring processes. The experiential aspect sustained by cinematic characters allows for grief processing in a safe and controlled environment that can foster post-traumatic growth. The last part of the therapy session was the creation of a therapeutic “sculturing”.

**Results:**

Family therapy clients’ accounts attested to their sense of safety in this setting, the re-enactment was described as non-traumatic. As the process advanced, they perceived progress from different stages of grief (frozen or depressed states) towards family healing.

As a further result, in the training group, the family therapists expressed a spontaneous recollection of previous personal losses. This was followed by a sense of relief and feelings of warmth and connection, mediated by both the creation of a safe holding space and by the end of session enactment of the sculpture.

**Conclusions:**

The model presented is a promising mixed therapy for addressing traumatic grief, that was well received and fostered personal and relational growth in a family therapy setting; as well as in psychotherapy training. Its design allows for further development and applications to group therapy.

**Disclosure of Interest:**

None Declared

